# Cognitive social capital and geriatric depression: A community-based case-control study among the rural elderly people of Bangladesh

**DOI:** 10.1017/gmh.2024.72

**Published:** 2024-10-22

**Authors:** Md. Ziaul Islam, Ely Prue, Sharmin Farjana, Md. Fuad Al Fidah, Syeda Sumaiya Efa

**Affiliations:** 1 Department of Community Medicine, National Institute of Preventive and Social Medicine (NIPSOM), Dhaka, Bangladesh; 2 Department of Community Medicine, Cox’s Bazar Medical College, Cox’s Bazar, Bangladesh; 3 Department of Reproductive Endocrinology and Infertility, Bangabandhu Sheikh Mujib Medical University (BSMMU), Dhaka, Bangladesh; 4 National Institute of Preventive and Social Medicine (NIPSOM), Dhaka, Bangladesh; 5 USAID’s ACTB, BADAS TB Initiative, Dhaka, Bangladesh

**Keywords:** cognitive, social capital, geriatric depression, rural older, interpersonal trust, reciprocity

## Abstract

**Background:**

Geriatric depression results in additional difficulties for older people and their residing society. The case-control study intended to assess the association between cognitive social capital and depression in rural older people.

**Methods:**

We conducted this study from January to December 2020 among 420 rural tenants aged ≥60 years in Bangladesh. We enrolled 210 older persons with depression as cases and another 210 without depression as controls. We used a semi-structured questionnaire, the Geriatric Depression Scale (GDS-15), and a cluster sampling technique to collect data through face-to-face interviews. We performed quality control checks and followed all ethics guidelines.

**Findings:**

Geriatric depression had a significant association with gender (*p* = 0.006), marital status (*p* < 0.001), education (*p* < 0.001), occupation (*p* = 0.001), family type (*p* < 0.001), family size (*p* < 0.001), number of family members (*p* < 0.001), and monthly family income (*p* < 0.001) of the rural older adults. Both interpersonal trust (*p* < 0.001) and reciprocity (*p* < 0.001) were significantly associated with geriatric depression. The older adults who didn’t believe in interpersonal trust (OR = 6.8, *p* = 0.002) and who disagreed with reciprocity (OR = 31.1, *p* < 0.001) were more likely to have depression.

**Implications:**

The study findings can contribute to formulating cognitive social capital policy and interventions to promote the psychological well-being of rural older people by alleviating geriatric depression.

## Impact statement

Population aging has been taking place rapidly in Bangladesh. The country has one of the fastest-growing aging populations in Southeast Asia. Geriatric depression has become a leading mental health problem among older adults, particularly in rural areas. This study examined the relationship between depression and cognitive social capital among rural older adults in Bangladesh. Geriatric depression was significantly associated with marital status, education, occupation, family type, family size, and family income of older individuals. Both the interpersonal trust and reciprocity domains of cognitive social capital were found to be associated with depression. Older persons without interpersonal trust had more chance of experiencing depression. Independence, autonomy, reciprocity, and trust of older individuals with their families and communities could foster cognitive social capital. The study findings could contribute to designing future social capital policies and interventions for the well-being of rural older people in a developing country like Bangladesh. Community-based motivational interventions could encourage older citizens to be involved in volunteering diverse social activities to prevent geriatric depression.

## Introduction

Aging is a process that begins at conception and continues throughout the lifespan. Considering the rapid increase in the number of older population, several instruments are adapted to assess the quality of life and physical resilience of older people. Different instruments are proposed to encompass their mental and physical capacities associated with functional ability, intrinsic capacity, and healthy aging. The most popular and validated instruments include the WHOQOL-BREF for quality of life (Lin et al., 2016), COSMIN for intrinsic capacity (Chen et al., 2023), and PRIFOR, and EQ5D for resilience (Fan et al., 2023; Hu et al., 2022). An increasing body of evidence suggests that psychological and sociological factors cause mental disabilities in older adults (Singh and Misra, 2009). Depression is an established mental disorder in older adults of both industrialized and developing nations. It is projected to become the leading cause of global illness burden by 2030 (Lépine and Briley, 2011). Due to its severe morbidity and mortality rates among older persons, depression is a leading public health concern. Depression is not a normal part of aging; it is a treatable medical illness, yet the risk of depression among older adults remains high. Geriatric depression is one of the most prevalent mental health issues worldwide. Depression was identified as the second leading cause of the global burden of mental illness in terms of DALYs estimated for all ages by 2020 (Reddy, 2010).

Geriatric depression results in additional difficulties for older persons and society, including a decrease in quality of life and functional capacity and an increase in using medical services and in the mortality rate (Vicerra, 2022; Chen et al., 2019a, 2019b). It also increases the incidence of comorbid dementia. The double burden of depression and dementia poses a huge burden on their family caregivers, which in turn contributes to the poor quality of life of family caregivers (Su et al., 2019, 2020; Hu et al., 2023). Populations are quickly aging; many countries face substantial challenges due to astounding rises in healthcare and long-term care expenses for older persons (Katon et al., 2003). Bangladesh is one of the twenty developing nations with the highest proportion of senior citizens. The country, along with four other Asian nations, will account for over half of the world’s old population by 2025 (Chaklader et al., 2003).

Today’s population is aging faster than ever before. From 2015 to 2050, the percentage of the world’s older population will nearly double from 12% to 22%. In 2050, eighty percent of older persons will reside in low- and middle-income countries. Approximately 15% of older adults suffer from mental diseases, while 10–20% of older individuals worldwide have depression (Cao et al., 2016; World Health Organization, 2001). In Asian countries, the prevalence of geriatric depression ranges from 12–34% (Wada et al., 2004). Over 7.5% of the country’s total population are senior citizens and will reach 20% by 2050 (Bangladesh Bureau of Statistics, 2015), and one out of every five Bangladeshi will be an older citizen. The prevalence of mental diseases ranged from 6.5% to 31.0% in Bangladesh and from 36.9% to 45% among the older population (Hossain et al., 2014). Rural residence, loneliness, illiteracy, and lack of religious practice and social support were associated with geriatric depression in Bangladesh (Disu et al., 2019).

Social capital has been addressed in diverse vantage points by experts. From the standpoint of social cohesion, social capital denotes “properties of social organization, such as trust, norms, and networks that can enhance the efficiency of society by promoting coordinated action” (Putnam et al., 1992). Social capital comprises two dimensions, including cognitive social capital, and structural social capital (Nyqvist et al., 2013). Cognitive social capital consists of the rules, values, and beliefs that affect the engagement of individuals in society (Agampodi et al., 2015) and is measured through neighborly trust and reciprocity (Nyqvist et al., 2013). Structural social capital depicts interactions between individuals frequently organized in formal community groups and measured by membership in organizations and social activities (Agampodi et al., 2015).

In Bangladesh, social support and mental health are intimately linked (Islam and Iqbal, 2008). Studies demonstrate that those with more social support have fewer mental health issues. Diverse management concerns like altering socioeconomic and demographic transitions, widespread poverty, shifting social and religious values, and inadequate health care facilities for the senior population exist in Bangladesh. Consequently, older persons suffer from numerous issues, including debt, loss of authority, social instability, inadequate leisure facilities, and a lack of comprehensive physical and mental care. All these make the older individuals more susceptible to depression.

The indicators for subjective well-being and life satisfaction of the older population include interpersonal trust and reciprocity. Socio-demographic transition, losing spouses, friends, social networks, social involvement, and support tend to predispose depression among older citizens. For this, the cognitive part of social capital is essential for day-to-day assistance and support for the older segment of the community. This case-control study intended to assess the relationship between cognitive social capital and depression in the rural older population.

## Methods

### Study settings

This case-control study was conducted from January to December 2020. We selected the Gobra union from the Sadar upazila of the Gopalganj district as the geographical area of interest. The study population included all the rural older individuals residing in rural communities of the Gobra Union. The Gobra union comprises nine wards having defined geographical boundaries.

### Sample size and sampling technique

**Figure 1.**
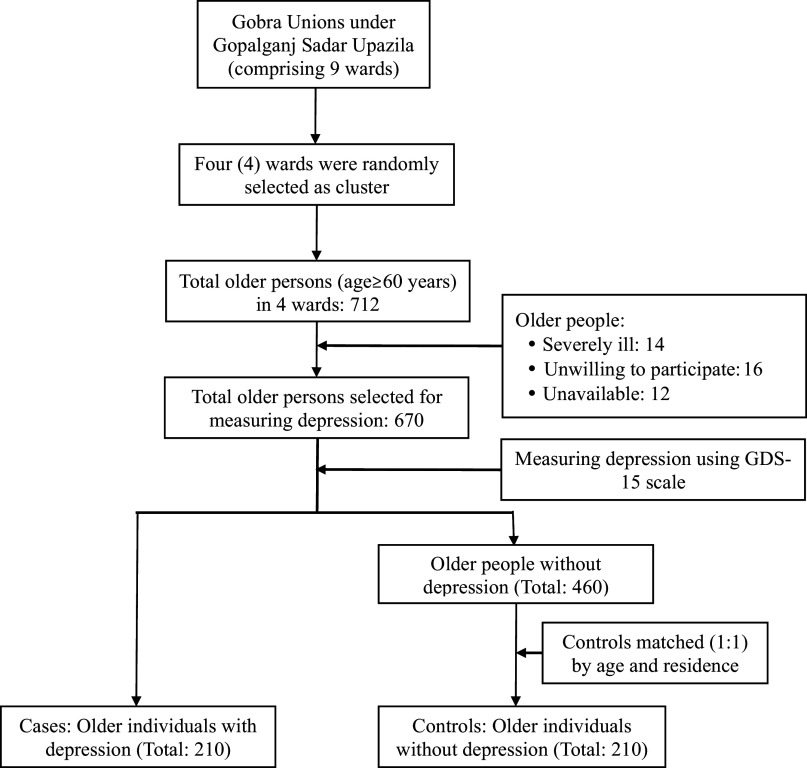
Flowchart of selecting study participants (Cases and Controls).

We calculated the sample size using the appropriate formula for a case-control study. The calculated sample size was 564 (284 cases and 284 controls). Considering the resource constraints and selection criteria, the study enrolled 420 older individuals, including 210 cases and 210 controls. We used a cluster sampling technique to recruit the participants from the rural communities. We considered each ward of the Gobra union with a defined geographical boundary as a cluster, and four wards were selected randomly as four clusters. Our field team (comprising two field supervisors and four data enumerators) visited all the households within the clusters (wards) and identified 712 households with older members. One eligible participant was selected from each household randomly. Among 712 identified rural older individuals, 14 (1.97%) were severely ill, 16 (2.24%) were unwilling to participate, and 12 (1.68%) were unavailable during data collection. Finally, 670 older persons were eligible for the study. The expert team (comprising a psychiatrist and a public health expert) used the Geriatric Depression Scale-15 (GDS-15) and identified 210 older individuals with depression for recruiting as cases and another 210 older persons without depression for recruiting as controls (Figure 1). To ensure adequate power, effect size was calculated for the chi-square test between the type of participants and outcome variables (Interpersonal trust and Reciprocity). For interpersonal trust, the effect size was 0.59, and for reciprocity, it was 0.81. Finally, we used G*Power (version 3.1) software to conduct the post hoc power analysis, which was found to be >0.80 in both cases (Kang, 2021).

### Study cases

Inclusion criteria:

1. Both male and female older persons aged 60 years and above.
2. Older persons suffering from depression are diagnosed by experts (comprising public health experts and psychiatrists) using the Geriatric Depression Scale-15 (GDS-15).
3. Older individuals who provided informed consent.

Exclusion criteria:

1. Older persons who were not willing to participate.
2. Older persons who were absent during data collection on two occasions.
3. Participants who were severely ill.

### Study controls

Inclusion criteria:

1. Both male and female older persons aged 60 years and above.
2. Older persons not suffering from depression were identified by experts (comprising public health experts and psychiatrists) using the Geriatric Depression Scale-15 (GDS-15).
3. Older individuals who provided informed consent.

Exclusion criteria:

1. Older persons who were not willing to participate.
2. Older persons who were absent during data collection on two occasions.

Controls were enrolled (ratio of 1:1 to cases) and matched individually to each case by age (±2 years) and residence (rural). We described the study objectives and procedure to all the controls and obtained informed consent for their participation. Due to different reasons like social and religious norms of rural communities, unavailability and unwillingness of some participants, scattered geographical distribution of rural households, work engagement of participants, and discontinuation of interviews, we couldn’t match gender to enroll the controls.

### Data collection methods and instrument

The study used a semi-structured questionnaire to collect data through face-to-face interviews. The questionnaire comprised variables related to background features (age, gender, marital status, educational qualification, occupation, family type, number of family members, monthly family income) and cognitive social capital (interpersonal trust, reciprocity). It also included the items of the GDS-15 checklist to determine geriatric depression. The present study used a Bangla version of the scale for collecting data related to depression, which was previously used by another study and was validated (Lahiri et al., 2020). The data enumerators made appointments with the participants to conduct the interviews at a convenient time and place. Each interview took approximately 25 minutes to complete.

### Operational definitions

#### Geriatric depression

The GDS-15 scale is widely used and has achieved validation in clinical and community settings for assessing depression among older individuals (Yesavage et al., 1982). There are several versions of GDS scales: GDS-15, a 5-item version by Hoyl MT et al., (1999) and Molly DW et al., (2006), and a 4-item version by Marwijk HWV et al., (1995), and Li YP et al., (2021). The current study used the GDS-15 scale to evaluate depression symptoms that had been present the week before. The scale consists of 15 items, which need answering with a “yes” or “no” answer. Out of 15 items, 10 indicate the presence of depression if positive answers are received. The remaining items (Items 1, 5, 7, 11, and 13) denote the presence of depression when answered negatively. The scale has a total score of 15 (ranging from 0 to 15). A cut-off score of ≥5 has been suggested to indicate depression and exhibits 92% sensitivity, and 89% specificity (Sheikh and Yesavage, 1986). The current study also considered a cut-off score of≥5. The internal consistency of the scale was quite good (Cronbach’s alpha = 0∙796) in the present study.

#### Elderly

People aged 60 years or above were considered elderly individuals.

#### Cognitive social capital

The cognitive dimension of social capital included interpersonal trust and reciprocity using a single-item questionnaire. The study assessed interpersonal trust by asking the participants an item: “Generally, do you think that most people are trustworthy?” This variable was coded with a 3-point Likert scale where “1” meant “Maximum people are trustworthy”; 2 meant “Try to be careful”, and “3” meant “I don’t know.” In logistic regression analysis, we graded interpersonal trust 1 = Believe (“Maximum people are trustworthy” and “Try to be careful”) and 2 = Not believe (“I don’t know”).

The study measured “Reciprocity” by using an item asking the participants: “Are you willing to help your neighbor who urgently needs your help [e.g. blood donation]?” We coded the variable with a 5-point Likert scale where “1” meaning “Strongly agree” and “5” meaning “Strongly disagree.” For logistic regression, we graded “Reciprocity” as “1” = Disagree (strongly disagree, disagree, neither agree and nor disagree) and “2” = Agree (agree, strongly agree). Several studies used these single-item questionnaires previously to evaluate interpersonal trust and reciprocity (Kim et al., 2017; Han and Lee, 2015; Park, 2017).

### Data management

Data were checked and verified after collection at the field and central level to ensure quality. Data were kept safely under the control of the principal investigator. We checked the data thoroughly to verify its relevancy and consistency. Data were coded, categorized, cleaned, and entered into software for analysis. We also performed a double entry of data for its quality control check.

### Statistical methods

The study used IBM SPSS software (Version 26.0) for data analysis. In the case of descriptive statistics, we estimated frequency distribution, percentage, mean, and standard deviation. Continuous data were tested by Shapiro-Wilk for normality and presented as mean and standard deviation. Categorical data were represented as counts and percentages. To find any association between categorical variables, we used the chi-square test as a part of inferential statistics. We developed a logistic regression model with all significant variables identified by chi-square tests to find the strength of association. A *p*-value <0.05 was considered statistically significant. All statistical tests were two-sided and performed at a significance level of α = 0.05.

## Results

A total of 420 older individuals (210 depressed as cases and 210 non-depressed as controls) participated in this case-control study. The study found no significant differences in age between cases and controls. Females were 55.2% among cases and 41.9% among controls, while males were 44.8% among cases and 58.1% among controls. Regarding marital status, most of the cases (64.8%) and controls (86.7%) were married, and this difference was statistically significant (*p* < 0.001). Concerning educational status, primary education was highest in the controls (49.5%), and illiterate was highest in the cases (65.7%), which was statistically significant (*p* < 0.001). Concerning occupation, housewife was significantly higher (*p* < 0.01) among cases (54.3%) than in the controls (41.0%). Joint family was found to be higher among the control (82.9%) than the cases (63.7%), which was statistically significant (*p* = 0.001).

Regarding the number of family members, there was a statistically significant (*p* < 0.001) difference between cases (53.3% had 5–8 members) and controls (65.7% had 5–8 members). In respect of monthly family income, the higher segment of the cases (56,2%) had 5000–10,000 taka, while the higher portion of the controls (56,2%) had 11,000–20,000 taka, and the difference was significant (*p* < 0.001) statistically (Table 1).

**Table 1.** Comparison of socio-demographic characteristics between cases and controls

Attributes		Type of participants		*p*-value
		Case, *f* (%)	Control, *f* (%)	
Age (in years)	60–69	134 (63.8)	146 (69.5)	0.441
	70–79	70 (33.3)	58 (27.6)	
	≥80	6 (2.9)	6 (2.9)	
	Mean ± SD	67.84 ± 4.79	67.34 ± 4.87	
Gender	Male	94 (44.8)	122 (58.1)	0.006
	Female	116 (55.2)	88 (41.9)	
Marital status	Married	136 (64.8)	182 (86.7)	<0.001
	Widow/widower	74 (35.2)	28 (13.3)	
Educational qualification	Illiterate	138 (65.7)	68 (32.4)	<0.001
	Primary	68 (32.4)	104 (49.5)	
	Secondary	4 (1.9)	38 (18.1)	
Occupation	Housewife	114 (54.3)	86 (41.0)	0.001
	Business	8 (3.8)	16 (7.6)	
	Service	0 (0.0)	12 (5.7)	
	Agriculture	58 (27.6)	52 (24.8)	
	Retired	30 (14.3)	44 (21.0)	
Family type	Nuclear	76 (36.2)	36 (17.1)	<0.001
	Joint	134 (63.8)	174 (82.9)	
Number of family members	2–4	78 (37.1)	38 (18.1)	<0.001
	5–8	112 (53.3)	138 (65.7)	
	9–12	20 (9.5)	34 (16.2)	
	Mean ± SD	5.38 ± 2.33	6.39 ± 2.34	
Monthly family income (Tk.)	5000–10,000	118 (56.2)	0 (0.00)	<0.001
	11,000–20,000	86 (41.0)	118 (56.2)	
	21,000–30,000	6 (2.9)	66 (31.4)	
	31,000–60,000	0 (0.0)	26 (12.4)	
	Mean ± SD	11,352.38 ± 5191.05	24,076.19 ± 11,225.99	

*f*: frequency; %: percentage; SD: standard deviation; significance: Chi-square test, *p* < 0.05 with 95% CI.

Both interpersonal trust and reciprocity were found to be significantly (*p* < 0.001) different between cases and controls. Regarding interpersonal trust, 55.2% of the cases answered, “I don’t believe,” while 71.4% of the controls answered, “Maximum people are trustworthy.” Concerning reciprocity, 45.7% of the cases answered “Disagree,” while 60.0% of the controls answered, “Strongly agree” (Table 2).

**Table 2.** Comparison of cognitive social capital (interpersonal trust and reciprocity) between cases and controls

Domains of cognitive social capital		Type of participants		*p*-value
		Case, *f* (%)	Control, *f* (%)	
Interpersonal trust	Maximum people are trustworthy	4 (1.9)	150 (71.4)	<0.001
	Try to be careful	90 (42.9)	56 (26.7)	
	I don’t believe	116 (55.2)	4 (1.9)	
Reciprocity	Strongly disagree	20 (9.5)	0 (0.0)	<0.001
	Disagree	96 (45.7)	2 (1.0)	
	Neither agree nor disagree	20 (9.5)	2 (1.0)	
	Agree	72 (34.3)	80 (38.1)	
	Strongly agree	2 (1.0)	126 (60.0)	

*f*: frequency; %: percentage; SD: standard deviation; *p* < 0.05: significant at 95% CI.

The logistic regression analysis showed that participants who didn’t have interpersonal trust were 6.771 times more at odds of experiencing depression than those who had interpersonal trust (AOR = 6.771; 95% CI: 1.985–23.094; *p* = 0.002). Concerning reciprocity, older individuals who didn’t agree to help neighbors who urgently needed help had more than 31 times higher risk of having depression (AOR = 31.133; 95% CI: 9.93–97.602; *p* < 0.001) (Table 3).

**Table 3.** Logistic regression analysis of the association between cognitive social capital (interpersonal trust and reciprocity) and depression

Domains of cognitive social capital		Regression coefficient(B)	OR	95%CI for OR		*p*-value
				Lower	Upper	
Interpersonal trust	Believe	Reference				
	Not believe	1.91	6.8	1.98	23.09	0.002
Reciprocity	Agree	Reference				
	Disagree	3.44	31.1	9.93	97.60	<0.001

OR: odds ratio: CI: confidence interval; reference category for depression: absent.

## Discussion

Social capital is a determinant of mental health and explains why mental health varies across societies. Lower social capital was connected to mental diseases such as depression and high suicide rates by previous research (Han and Lee, 2015; Bassett and Moore, 2013). Hence, it is a social element of importance for public health, notably for older persons and healthy aging (Aihara et al., 2009). Literature also suggests that interpersonal trust and reciprocity are crucial determinants of older mental health characteristics, and they affect life satisfaction, self-rated health, depression, physical disability, and death rates in older adults (Lu et al., 2018; Islam, 2019). The current study examined the relationship between social capital and depression in rural older adults in Bangladesh.

Previous literature review revealed that relevant data on cognitive social capital and geriatric depression is not available in the context of rural Bangladesh. This case-control study was conducted between two groups of older population, cases having depression and controls having no depression. Using a community-based case-control study design, the current study sought to uncover the link between cognitive social capital and older adults` depression in a rural setting.

The present study found a statistically significant association between gender and depression among older individuals, which collaborates with previous studies that have also found the same findings (Zhou et al., 2022; Konda et al., 2018). This may be because older women are often deprived of adequate care, more so than their counterparts in the sub-continent. The marital status of older people has a significant association with geriatric depression. Another study conducted in India also reports similar findings (Konda et al., 2018). This may be because older people are culturally dependent on their spouses for care and emotional support, a lack of which often leads to depression. A statistically significant association was also found between educational qualification, occupation, family type, number of family members, and monthly family income (in taka) with depression in older adults.

In this study, it was found that a higher level of cognitive social capital (in terms of interpersonal trust and reciprocity) was associated with a lower chance of having depressive symptoms, which corresponds to previous studies (Han et al., 2018; Li et al., 2017; Cao et al., 2015). Previous studies also suggested that a lower level of cognitive and social capital (in terms of interpersonal trust and reciprocity) is associated with suffering from depression among older people, which conforms with our study. These findings indicate the importance of cognitive social capital while addressing the issues of depression among older people. Several studies have suggested some methods that can help to improve the level of cognitive social capital among older people which will consequently improve the mental health condition and quality of life of older adults. Using social networks is an adaptive method that can help older people to overcome their depression and increase the quality of life (Siah et al., 2023). Implementing physical activity programs and promotion of vaccination programs can also improve the quality of life of older individuals (Lee et al., 2024; Lin et al., 2022).

## Conclusion

The present study provided evidence on the relationship between cognitive social capital and geriatric depression and showed that cognitive social capital is associated with depression. Individuals who had lower reciprocity and trust were more prone to have depression. The literature review revealed that relevant data on cognitive social capital and geriatric depression is scarce in the context of rural Bangladesh. The findings could be helpful in developing policies and programs for public awareness to promote and maintain social capital activities to safeguard our older people from late-life depression.

### Limitations of the study

The study enrolled only older people from the rural communities of Bangladesh. So, the study findings could not be generalized to the whole country. In addition, for some valid reasons, the study could not match gender in enrolling the control group.

### Recommendations

Depression among older persons in rural areas needs to be an integral component of public health interventions in the community. To prevent depression, reciprocal relationships, and social interaction of Older with their family, friends, and neighbors should be increased. Interpersonal trust needs to be ensured within families and communities to prevent older depression. To provide need-based interventions for the prevention and control of depression among rural elderly individuals, community-based activities like night education, age-based vocational training, etc., should be required.

## Data Availability

The data that support the findings are available on request from the corresponding author, MZ Islam. The data are not publicly available due to containing information that could compromise the privacy of research participants.
