# Supplementary material for: Cognitive social capital and geriatric depression: A community-based case-control study among the rural elderly people of Bangladesh
Source: Glob Ment Health (Camb). 2024 Oct 22;11:e89. doi: 10.1017/gmh.2024.72 (PMC11504949; doi:10.1017/gmh.2024.72)
Supplement: Peer review history

# Editor report: Recommendation: Cognitive social capital and geriatric depression: A community-based case-control study among the rural elderly people of Bangladesh — R0/PR2

**DOI:** 10.1017/gmh.2024.72.pr2

**Affiliations:** University of Zimbabwe Faculty of Medicine, Zimbabwe

Thank you for submitting your manuscript.

In the abstract section instead of “..maintained ethical issues properly” please edit this to "we followed all ethics guidelines'.

In the introduction section please include more recent citations from the past 5 years or so that show the landscape of depression in older patients and cognitive social capital in recent years.

# Editor report: Decision: Cognitive social capital and geriatric depression: A community-based case-control study among the rural elderly people of Bangladesh — R0/PR3

**DOI:** 10.1017/gmh.2024.72.pr3

**Affiliations:** London School of Hygiene & Tropical Medicine, United Kingdom of Great Britain and Northern Ireland

No accompanying comment.

# Editor report: Recommendation: Cognitive social capital and geriatric depression: A community-based case-control study among the rural elderly people of Bangladesh — R1/PR5

**DOI:** 10.1017/gmh.2024.72.pr5

**Affiliations:** University of Zimbabwe Faculty of Medicine, Zimbabwe

No accompanying comment.

# Editor report: Decision: Cognitive social capital and geriatric depression: A community-based case-control study among the rural elderly people of Bangladesh — R1/PR6

**DOI:** 10.1017/gmh.2024.72.pr6

**Affiliations:** London School of Hygiene & Tropical Medicine, United Kingdom of Great Britain and Northern Ireland

No accompanying comment.

# Editor report: Recommendation: Cognitive social capital and geriatric depression: A community-based case-control study among the rural elderly people of Bangladesh — R2/PR8

**DOI:** 10.1017/gmh.2024.72.pr8

**Affiliations:** University of Zimbabwe Faculty of Medicine, Zimbabwe

No accompanying comment.

# Editor report: Decision: Cognitive social capital and geriatric depression: A community-based case-control study among the rural elderly people of Bangladesh — R2/PR9

**DOI:** 10.1017/gmh.2024.72.pr9

**Affiliations:** London School of Hygiene & Tropical Medicine, United Kingdom of Great Britain and Northern Ireland

No accompanying comment.
